# Identification of miR-499a-5p as a Potential Novel Biomarker for Risk Stratification in Endometrial Cancer

**DOI:** 10.3389/fonc.2021.757678

**Published:** 2021-10-29

**Authors:** Gloria Ravegnini, Antonio De Leo, Camelia Coada, Francesca Gorini, Dario de Biase, Claudio Ceccarelli, Giulia Dondi, Marco Tesei, Eugenia De Crescenzo, Donatella Santini, Angelo Gianluca Corradini, Giovanni Tallini, Patrizia Hrelia, Pierandrea De Iaco, Sabrina Angelini, Anna Myriam Perrone

**Affiliations:** ^1^ Department of Pharmacy and Biotechnology (FaBiT), University of Bologna, Bologna, Italy; ^2^ Centro di Studio e Ricerca delle Neoplasie Ginecologiche (CSR), University of Bologna, Bologna, Italy; ^3^ Department of Experimental, Diagnostic and Specialty Medicine, Alma Mater Studiorum-University of Bologna, Bologna, Italy; ^4^ Molecular Pathology Laboratory, Istituto di Ricovero e Cura a Carattere Scientifico (IRCCS) Azienda Ospedaliero-Universitaria di Bologna/Azienda USL di Bologna, Bologna, Italy; ^5^ Department of Medical and Surgical Sciences (DIMEC), University of Bologna, Bologna, Italy; ^6^ Center for Applied Biomedical Research, Alma Mater Studiorum-University of Bologna, Bologna, Italy; ^7^ Division of Oncologic Gynecology Unit, IRCCS—Azienda Ospedaliero-Universitaria di Bologna, Bologna, Italy; ^8^ Pathology Unit, IRCCS Azienda Ospedaliero—Universitaria di Bologna, Bologna, Italy

**Keywords:** endometrial cancer, miRNA—microRNA, MMRd, TCGA, prognostic biomarkers, personalized medicine, NSMP

## Abstract

**Introduction:**

The Cancer Genome Atlas (TCGA) project identified four distinct prognostic groups in endometrial cancer (EC), among which two are correlated with an intermediate prognosis: the MisMatch Repair-deficient (MMRd) and the No Specific Molecular Profile (NSMP) groups. The two groups represent a heterogeneous subset of patients frequently harboring CTNNB1 alterations with distinctive clinicopathologic features. The study aimed to evaluate the miRNA expression in ECs to identify potential biomarkers of prognosis.

**Methods:**

We analyzed miRNA expression in 72 ECs classified as MMRd or NSMP including 15 ECs with CTNNB1 mutations. In the discovery step, miRNA expression was evaluated in 30 cases through TaqMan miRNA arrays. Subsequently, four miRNAs were validated in the total cohort of ECs. The data were further tested in the TCGA cohort, and correlations with overall survival (OS) and progression-free interval (PFI) were evaluated.

**Results:**

miR-499a-3p and miR-499a-5p resulted to be overexpressed in CTNNB1 mutant EC patients at intermediate risk. Similarly, in the TCGA cohort, miR-499a-3p and miR-499a-5p were differentially expressed between CTNNB1 mutant and wild-type patients (p < 0.0001). NSMP patients with low miR-499a-5p expression showed longer OS (p = 0.03, log-rank test). By combining miR-499a-3p or -5p expression levels with the CTNNB1 status, ECs with CTNNB1 mutation and lower miR-499a-5p expression showed better OS compared with the other subgroups (p = 0.03, log-rank test), among the NSMP patients. Moreover, in a multivariate analysis, combination of wild type CTNNB1 status and high miR-499a-5p expression was indipendently associated with high risk of death [HR (95%CI): 3.53 (1.1-10.5), p = 0.02].

**Conclusion:**

Our results suggest that the combination of CTNNB1 status and miR-499a-5p allows a better stratification of NSMP patients and could promote a personalization of the treatment in intermediate-risk patients.

## Introduction

Endometrial cancer (EC) is the most common gynecological cancer, with an increasing incidence in Western countries ranging between 15 and 25 per 100,000 women (1, 2).

Historically, three distinct but overlapping EC classifications have been proposed to define prognosis (3): pathogenetic, histopathological, and molecular classification. However, despite their merits, the three classifications are not able to uniquely define the complexity and heterogeneity of the disease and present various issues. Histopathological parameters used alone to identify risk factors are not always easily reproducible, particularly in high-grade carcinomas and those with intratumoral heterogeneity (4–8).

To overcome this kind of problems, recently, The Cancer Genome Atlas (TCGA) endometrial collaborative project identified four distinct prognostic EC groups based on molecular alterations: (i) the ultramutated subtype that encompassed POLE exonuclease domain mutated (POLE) cases (excellent prognosis); (ii) the hypermutated subtype, characterized by MisMatch Repair deficiency (MMRd) (intermediate prognosis); (iii) the copy-number high subtype, with p53 abnormal/mutated features (p53abn) (poor prognosis); and (iv) the copy-number low subtype, also known as No Specific Molecular Profile (NSMP) (intermediate prognosis) (9). However, in order to translate the proposed TCGA scheme into clinical practice, two independent research groups proposed and validated surrogate markers (POLE mutation, microsatellite instability, and p53abn to improve the integration of the TCGA classification in the routine clinical practice (9–12). In 2021, the European Society of Gynecological Oncology (ESGO), the European Society for Radiotherapy and Oncology (ESTRO), and the European Society of Pathology (ESP) published updated guidelines for the determination of the risk group in EC, integrating both molecular and clinicopathological diagnostic variables, with the aim of improving patients’ treatment (13).

The ESGO/ESTRO/ESP guidelines consider all POLE mutant ECs up to stage II to be at low risk of recurrence regardless of other ESMO risk parameters, and adjuvant treatments are deemed unnecessary in these cases. The p53abn group benefits from adjuvant therapy which is also expected to be aggressive independently of histology and different managements for these two entities may not be recommended. Within the MMRd and NSMP groups, which have an intermediate prognosis, FIGO grade, LVSI, and depth of myometrial invasion are critical factors for risk stratification. Thus, in these two groups, treatment continues to rely largely on conventional risk factors, and the choice of the most appropriate adjuvant therapies remains a challenge. MMRd ECs represent a heterogeneous group of cancers. A better stratification within this group to select therapies is needed in consideration that a subgroup of MMRd ECs exhibiting MLH1 promoter methylation presents a worse prognosis than other MMRd ECs (14). In the NSMP group, the absence of specific molecular signatures appears to be accompanied by a more heterogeneous biological behavior than the other TCGA groups. This group mainly includes low-grade endometrioid-type ECs characterized by alterations in PI3K/AKT and Wnt/β-catenin signaling pathways often with mutations on exon 3 of CTNNB1 (52%). Although a few studies have characterized the CTNNB1 alterations in EC (15, 16), its prognostic significance among NSMP ECs is not completely understood; moreover, it is not clear if CTNNB1 mutations could indicate a distinct molecular group (17). This shows that integration of different prognostic factors may serve to accurately define the risk classes, leading to a more precise characterization of ECs which are extremely heterogeneous and sometimes unclassifiable.

MicroRNAs (miRNAs) are small non-coding RNAs spanning between 18 and 25 nucleotides in length, which are able to regulate specific target genes at the posttranscriptional level by inhibiting their expression (18). Consequently, miRNAs deregulation can have a deep impact on key cellular processes which, in turn, could drive carcinogenesis or promote cancer progression. In recent years, many studies investigating miRNA expression level in EC have been published, shedding light on this heterogeneous disease (19). However, identifying novel potential diagnostic biomarkers remains an unmet clinical need, particularly within the EC patients with intermediate prognosis who represent the most difficult category to manage from a clinical point of view (i.e., NSMP and MMRd groups).

Based on the above, we aimed to characterize the miRNA expression level in these groups defined as at intermediate risk, considering the CTNNB1 status, in order to better stratify these patients. To do that, we first focused on the NSMP and MMRd EC patients comparing patients harboring, or not, CTNNB1 mutations; secondly, we tested our findings in the TCGA EC cohort to corroborate them.

## Material and Methods

### Tumor Specimen Collection

Starting from a cohort of 117 consecutive surgical EC specimens characterized and divided into four groups according to the molecular classification, we selected the ones identified as NSMP or MMRd. Formalin-fixed, paraffin-embedded (FFPE) primary tumors from 72 EC patients were included in the study. Tumor samples were collected at the time of hysterectomy for EC; patients submitted to previous radiotherapy or chemotherapy treatment were excluded. FFPE specimens were retrieved from the archives of the departments of pathology of the IRCCS Azienda Ospedaliero-Universitaria of Bologna between 2014 and 2020. The selected blocks were used to assess histopathologic parameters, for immunohistochemical and molecular analyses, and were classified according to standard histopathologic criteria following the World Health Organization classification of tumors (20). The study was approved by the Institutional Review Board 189/2021/Oss/AOUBo, ClinicalTrials.gov Identifier: NCT04845425. **Supplementary Table S1** summarizes the main clinical and pathological features of the patients.

### Tissue Processing

All FFPE specimens were conserved in the Institution’s pathology archives, and two expert pathologists examined tissue slides to confirm the EC diagnosis and to ensure the inclusion of more than 70% of cancer cells. When the percentage was not respected, tissue slides were macro-dissected to eliminate contamination of non-tumoral components and guarantee that at least 70% of the samples for the analysis were tumor cells indicated by the pathologist. RNA was isolated from FFPE (three to six sections >10 µm for each sample) by RecoverAll Total Nucleic Acid Isolation Kit (Ambion, Thermo Scientific) following the manufacturer’s instructions. RNA integrity and quantification were evaluated using the 2100 Agilent Bioanalyzer. Mutation analysis was carried out as previously described (17).

### MiRNA Analysis

MiRNA expression was firstly evaluated in a discovery step comprising 30 ECs (discovery cohort), and the results were then validated in 72 ECs (n = 30 ECs overlapping with the discovery step + n = 42 new ECs, validation cohort).

### Discovery Step: miRNA Expression Profiling

The expression profiles of 384 miRNAs were analyzed in 30 primary tumors, of which 12 were NSMP CTNNB1 mutant (CTNNB1^mut^), 11 NSMP CTNNB1- wild-type (CTNNB1^wt^), and 7 MMRd. One nanogram of total RNA was reverse transcribed to cDNA, using TaqMan Advanced miRNA cDNA Synthesis Kit (Applied Biosystems, Thermo Scientific), which is specific for the detection and quantification of mature human miRNAs in biological samples. The cDNAs were then amplified using the Universal miR-Amp Primers and Master Mix to uniformly increase the amount of cDNA for each target, maintaining the relative differential expression levels. The cDNA was loaded into the TaqMan Array Advanced miRNA array pool A and run in a 7900HT Fast PCR System (Applied Biosystems).

### Validation of the Profiling Results by qRT-PCR

miRNA expression levels of miR-187-3p (assay ID # 477941_mir), miR-325 (assay ID # 478025_mir), miR-499a-3p (assay ID # 478948_mir), and miR-499a-5p (assay ID # 478139_mir) were evaluated by qRT_PCR through TaqMan Advanced miRNA Assay (Thermo Fisher Scientific). miR-16-5p (assay ID # 477860_mir, Thermo Fisher Scientific) was used as the internal reference (21), after the literature review and assessment of its stability in our study cohort. The analysis was conducted in all the 72 EC patients. miRNA expression was evaluated according to standard TaqMan Advanced miRNA assay protocol and run in a 7900HT Fast PCR System (Applied Biosystems). Each TaqMan Advanced miRNA assay was run in triplicate.

### Statistical Analysis

miRNA data were analyzed with SDS RQ Software version 2.4 and with a Thermo Fisher Cloud app (Thermo Fisher Scientific); miRNAs with Ct values ≥ 38 were considered as not expressed and excluded from further analysis. The relative expression levels were quantified using the 2^-ΔΔCt^ method using miR-16-5p as reference (22). Statistical significance was estimated using the non-parametric Mann–Whitney–Wilcoxon test. This non-parametric test allows problems related to equal variance, normality assumption, or those regarding the use of frequencies to be obviated. A p-value < 0.05 was considered statistically significant.

### Validation of the Results in the TCGA Cohort

MAF files for the UCEC TCGA project were downloaded and explored using the R (version 4.1.0) packages TCGAbiolinks and maftools. The selection of pathogenic CTNNB1 and p53 mutations was done considering the pathogenicity prediction by both PolyPhen and SIFT scoring systems. UCEC-curated molecular subtypes derived from the TCGA marker paper were retrieved from synapse through TCGAbiolinks (9, 23, 24). For expression analysis, sample-level log2 miRSeq and mRNASeq expression values were retrieved using the FireBrowse RESTful API (25). For clinical variables, the progression-free interval (PFI) and overall survival (OS) were used as outcome variables, as recommended by the PanCanAtlas Publications NCI Genomic Data Commons guidelines (https://www.cell.com/cell/fulltext/S0092-8674(18)30229-0). The NSMP EC subgroup was selected by the exclusion of POLE, MMRd, and p53-mutated tumors.

For each miRNA analyzed, the cohort of UCEC patients was divided into two groups based on the expression level of the miRNA of interest using the median expression value as a threshold. Statistical analysis was carried out using the GraphPad Prism 8 and SPSS v20 software. Comparisons between groups were conducted using the two-tailed Student’s t-test. Kaplan–Meier curves were plotted for the OS and PFI while the statistical significance was assessed using the log-rank test. For multivariate analysis, the COX proportional hazards model was used. A p value <0.05 was considered statistically significant.

The general workflow of the paper is presented in **Supplementary Figure 1**.

## Results

### Discovery Step: miRNA Expression Profiling

We analyzed miRNA expression profiling in the NSMP group (n = 23) comparing the CTNNB1 mutant (n = 12) and the CTNNB1 wild-type (n = 11) cases.

This comparison highlighted a significant upregulation of two miRNAs, namely, miR-499a-3p (p = 0.0002; padj = 0.023) and miR-499a-5p (p = 0.00009; padj = 0.013) in the CTNNB1^mut^ subgroup.

Subsequently, given the similar intermediate prognosis usually observed in MMRd ECs, we included seven consecutive additional MMRd cases, all CTNNB1^wt^, in our discovery step.

miRNA expression profiling was thus evaluated on a total of 30 cases with intermediate risk, of which 12 were NSMP CTNNB1^mut^, 11 NSMP CTNNB1^wt^, and 7 MMRd. We compared the cases with CTNNB1 mutation (n = 12) with the remaining cases, irrespective of NSMP or MMRd molecular group (n = 18). The results showed that 39 miRNAs presented a statistically significant deregulation, as reported in **Supplementary Table S2**. The top two deregulated miRNAs were again miR-499a-3p and miR-499a-5p.

The miRNet tool was used to perform pathway-enriched analysis; the results are reported in **Supplementary Table S3**.

### Validation of the Profiling Results by qRT-PCR

**Figure 1:**
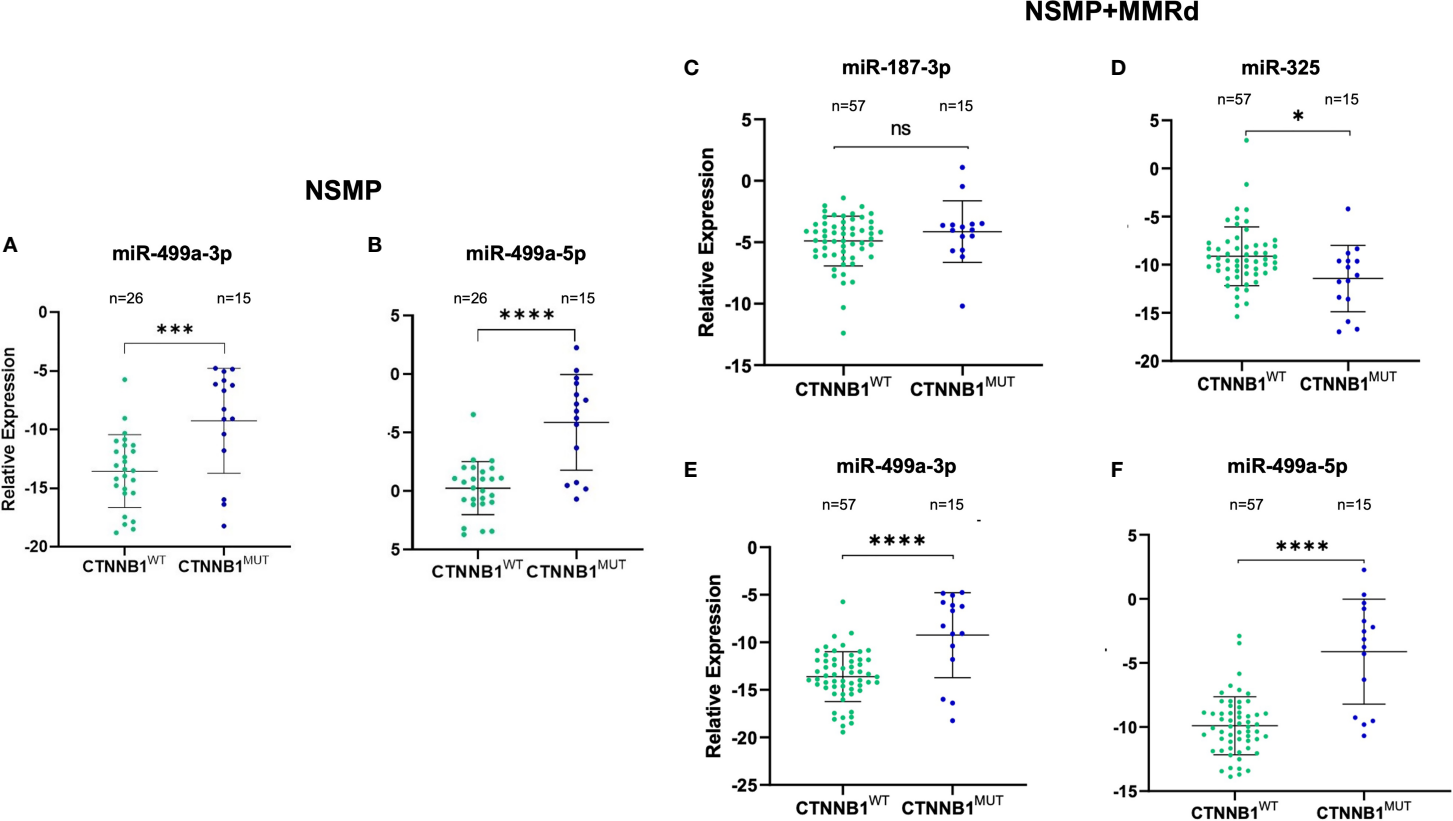
Validation of miR-499a-3p and 499a-5p in the NSMP ECs (n = 41) **(A, B)**, validation of miR-187-3p **(C)**, miR-325 **(D)**, miR-499a-3p **(E)**, and 499a-5p **(F)** in the 72 EC samples (NSMP + MMRd). ns, not significant (p > 0.05); *p < 0.05; ***p < 0.001; ****p < 0.0001.

Based on the p-value and on Ampscore, - a value released by the Thermo Fisher Cloud app which evaluates the goodness of RT-PCR amplification, - we selected four miRNAs to validate in 72 samples. Validation was performed in the NSMP cohort alone as well as in the NSMP + MMRd cohort. **Figure 1** shows the results.

In the NSMP ECs, miR-499a-3p and miR-499a-5p maintained the statistical significance between CTNNB1^wt^ and CTNNB1^mut^ patients (miR-499a-3p, p = 0.0008; miR-499a-5p p < 0.0001; **Figure 1**). As expected, based on the profiling data, miR-187-3p and miR-325 were not significantly deregulated between the two subgroups.

Regarding the extended cohort including NSMP and MMRd patients together, miR-187-3p did not maintain the statistical significance, whereas the other three miRNAs confirmed the differential expression between EC samples harboring the CTNNB1 mutation and the wild-type ones. In particular, miR-325 was downregulated in the CTNNB1^mut^ ECs compared with the CTNNB1^wt^ cases (p = 0.012), whereas miR-499a-3p and miR-499a-5p were both upregulated in CTNNB1^mut^ ECs with respect to CTNNB1^wt^ patients (p < 0.0001 for both). No association between miRNA expression and clinical parameters reported in **Table 1** was observed.

**Table 1:** Characteristics of the study cohort.

	N = 72 (%)
**Age**	
<50	6 (8.3)
>50	66 (91.7)
**BMI**	
<25	25 (34.7)
>25	47 (65.3)
**Histotype**	
E	62 (86.1)
I-DED	8 (11.1)
S	2 (2.8)
CS	0
CCC	0
**ESMO risk**	
Low risk	14 (19.4)
Intermediate risk	5 (6.9)
High-intermediate risk	31 (43.1)
High risk	22 (30.6)
**Grading**	
Low	55 (76.4)
High	17 (23.6)
**FIGO stage**	
IA	45 (62.5)
IB–II	13 (18.1)
III–IV	14 (19.4)
**LVI**	
Absent	25 (34.72)
Focal	23 (31.94)
Massive	23 (31.94)
Missing	1 (1.39)
**TCGA classification**	
NSMP	41 (56.9)
MSI	31 (43.9)

BMI, body mass index; E, endometrioid; I-DED, de-differentiated; S, serous; CS, carcinosarcoma; CCC, clear cell carcinoma; LVI, lymphovascular invasion.

### Validation of the Results in the TCGA EC Cohort

**Figure 2:**
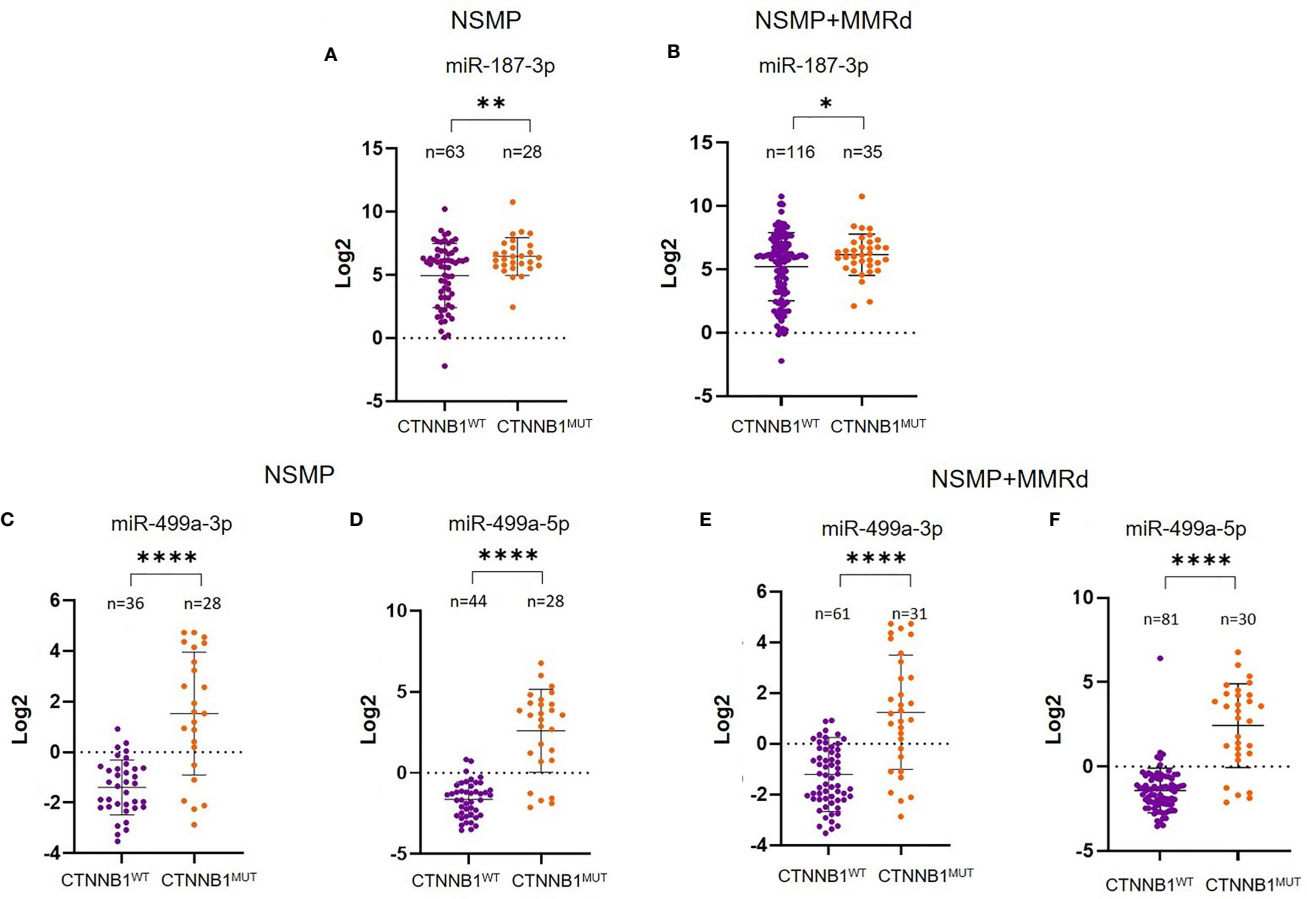
Analysis of miR-187-3p in the NSMP alone and in NSMP + MMRd TCGA cohorts **(A, B)**. Analysis of miR-499a-3p and miR-499a-5p in the TCGA NSMP alone **(C, D)** and in NSMP + MMRd EC groups **(E, F)**. *p < 0.05; **p < 0.01; ****p < 0.0001.

**Figure 3:**
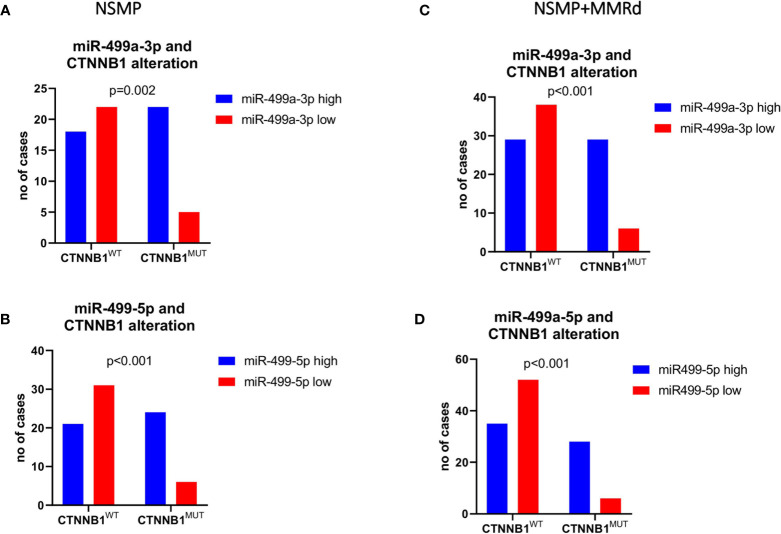
Chi-square test was used to test the distribution of low/high miR-499a-3p **(A, C)** and 5p **(B, D)** expression across the CTNNB1-mutated *versus* wild-type patients’ subgroups.

To corroborate our data, we further validated the results in an independent cohort of ECs. To do that, we analyzed the TCGA cohort of EC, stratifying the patients based on the CTNNB1 mutation. We were able to retrieve the data for 151 EC cases, of which 91 were NSMP and 60 MMRd. Of note, out of all miRNAs, miR-325 expression in the TCGA ECs could not be assessed. As we did for our cohort, we first analyzed miRNA expression considering only the NSMP cases, and subsequently, we included the MMRd cases. As shown in **Figures 2A, B**, miR-187-3p resulted to be significantly deregulated in both NSMP cohort alone and including the MMRd (p = 0.004 and p = 0.049, respectively). With regard to miR-499a-3p and -5p, the statistical significance was maintained in both the cohorts analyzed (**Figures 2C–F**). The chi-square test was used to assess the distribution of high/low miRNA expression based on the CTNNB1 mutational status (**Figure 3**). As clearly shown, the CTNNB1 mutant ECs presented a significant enrichment of tumors with high expression of the two miRNAs in NSMP alone and in NSMP+ MMRd cohorts.

### Prognostic Impact of miR-499a on TCGA EC Cohort

**Figure 4:**
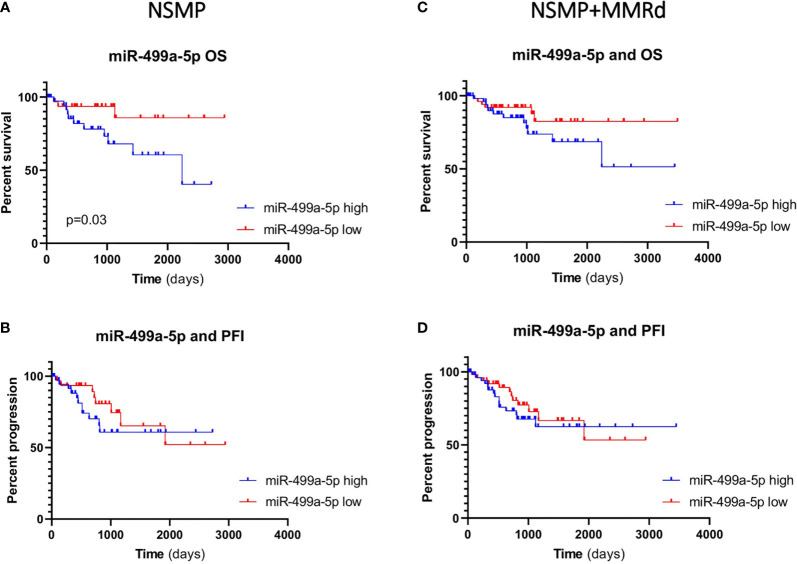
Kaplan–Meier estimates of overall survival **(A, C)** and progression-free interval **(B, D)** in NSMP alone and NSMP + MMRd groups, based on miR-499a-5p expression.

Given the small sample size of our study group and the limited number of events, we evaluated a possible association between miR-187-3p, miR-499a and OS and PFI in the TCGA EC cohort. First, we considered the expression of the two miRNAs in both NSMP and NSMP + MMRd cohorts. Based on miRNA expression, we divided the patients in two groups (high and low expression) using the median value of the specific miRNA as a cutoff. No significant associations were identified with miR-187-3p. The median values for miR-499a-3p and -5p were -1.19 and -1.25, respectively. Interestingly, miR-499a-5p was significantly associated with OS in the NSMP group (p=0.03, log-rank test). In particular, ECs with a lower miR-499a-5p expression showed better OS when compared to the patients with higher levels (**Figure 4**). In order to exclude other factors which may influence OS in the NSMP subgroup and to strengthen the prognostic value of miR499a-5p, we considered other clinical factors which can have an impact on patient outcome, such as tumor stage and grade. The COX proportional hazards model was used, and variables reaching significance in the univariate analysis were included in the multivariate model. Both high level of miR499a-5p [HR (95% CI): 3.96 (1.1–14.7), p = 0.04] and high tumor grade [HR (95% CI): 5.7 (1.7–18.9), p = 0.004] were independent predictors of poor survival in EC patients.

In the NSMP+ MMRd cohort, the same trend was observed, but without reaching a statistical significance (p = 0.13). PFI was not associated with miR-499a-5p expression neither in the NSMP group (p = 0.39) nor in the NSMP + MMRd (p = 0.49) group. MiR-499a-3p did not show any significant correlation (**Supplementary Figure 2**).

**Figure 5:**
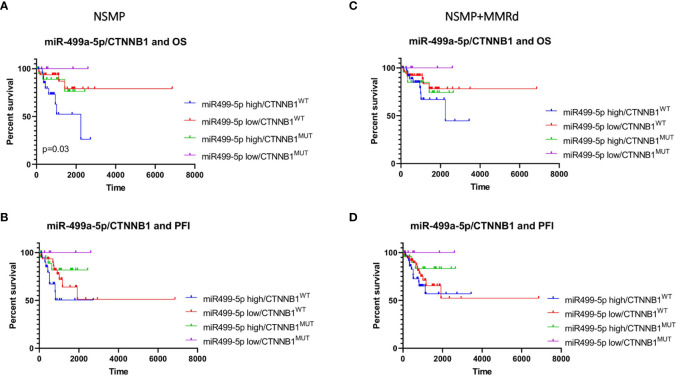
Kaplan–Meier estimates of OS **(A, C)** and PFI **(B, D)** in NSMP alone and NSMP + MMRd groups, based on the combination of miR-499a-5p expression and CTNNB1 status.

Finally, we combined miR-499a-3p and -5p expression levels (low or high) with the CTNNB1 mutational status (mutant or wt). The combination of miR-499a-3p and CTNNB1 status did not reveal any significant correlation with OS and PFI in NSMP alone or NSMP + MMRd cohorts (**Supplementary Figure 3**). On the contrary, CTNNB1 mutant ECs with lower miR-499a-5p expression showed better OS compared with the other subgroups, among the NSMP patients (**Figure 5**). Conversely, the combination of miR-499a-5p expression and CTNNB1 mutational status showed a significant difference in OS in the NSMP patients (p=0.03, log-rank test) (**Figure 5**). Specifically, ECs with no CTNNB1 mutations and high miR-499a-5p expression, showed shorter OS compared with the other groups [HR (95% CI): 3.99 (1.3–11.6), p = 0.006]. Moreover, in a multivariate analysis, the same combination of miRNA expression and CTNNB1 mutational status remained independently associated with higher risk of death [HR (95% CI): 3.53 (1.1–10.5), p = 0.02]. The same trend was observed in NSMP + MMRd patients, but without reaching statistical significance (p = 0.37). With regard to the PFI, even if no significant correlations were observed, CTNNB1 mutant ECs with low 499a-5p expression showed better PFI in NSMP alone and NSMP + MMRd combined groups (p = 0.14 and p = 0.26, respectively). The combination of miR-499a-5p and CTNNB1 mutational status allows for a better stratification of EC patients with respect to the CTNNB1 mutation alone (**Supplementary Figure 4**).

## Discussion

In the last decade, several miRNAs have been described as potential biomarkers of EC prognosis or diagnosis. For example, among others, miR-34a was identified as able to stratify patients at high risk of recurrence (26) and reduced miR-497-5p levels were reported in high-grade ECs compared with low-grade Ecs (27). However, to the best of our knowledge, this is the first study to analyze miRNA expression in EC with the aim to obtain prognostic information in the intermediate-risk groups (i.e., NSMP and MMRd) of the molecular classification, trying to create subclassifications while taking into account the CTNNB1 mutational status.

In the attempt to better define this latter group, we first evaluated the miRNA expression profile in CTNNB1^mut^ ECs compared with the CTNNB1^wt^ NSMP ones. To this purpose, we analyzed the miRNA expression profile in 23 EC cases, identifying miR-499a-3p and 5p as significantly upregulated miRNAs in the CTNNB1^mut^ cases when compared with the wt ones. However, considering that in literature the NSMP EC subgroup appears to be similar to the MMRd group at the prognosis level, we included seven additional MMRd patients.

Based on the results, we selected four miRNAs to validate in 72 EC samples and we observed a high statistical significance for miR-499a-3p and -5p. Finally, to corroborate our data, we analyzed the TCGA EC cohort, stratifying the patients based on the CTNNB1 mutation. In agreement with our results, the CTNNB1^mut^ patients showed a higher expression of both miR-499a-3p and -5p in NSMP ECs as well as among the NSMP/MMRd patients. We further analyzed the survival rate in terms of OS and PFI with regard to the two-miRNA level. We did not perform this analysis in our study cohort due to the limited number of samples and events which, from a statistical point of view, will not give strong results. In fact, considering the MSI and NSMP groups, the time needed for the development of such events is a limiting factor and requires a statistical power that we did not have; therefore, at this time, validation of our results in the TCGA cohort appeared more appropriate.

Based on miRNA expression, the patients were divided into two groups (high and low expression) using the median of their expression level as a cutoff. MiR-499a-5p was significantly associated with OS, showing that NSMP patients with a lower expression have a prolonged survival. Conversely, the high level of miR-499a-5p was an independent predictor of poor survival.

The same correlation was observed when miRNA level and CTNNB1 status were combined. Indeed, NSMP patients with no CTNNB1 mutations and high miR-499a-5p expression had an increased risk of death.

**Figure 6:**
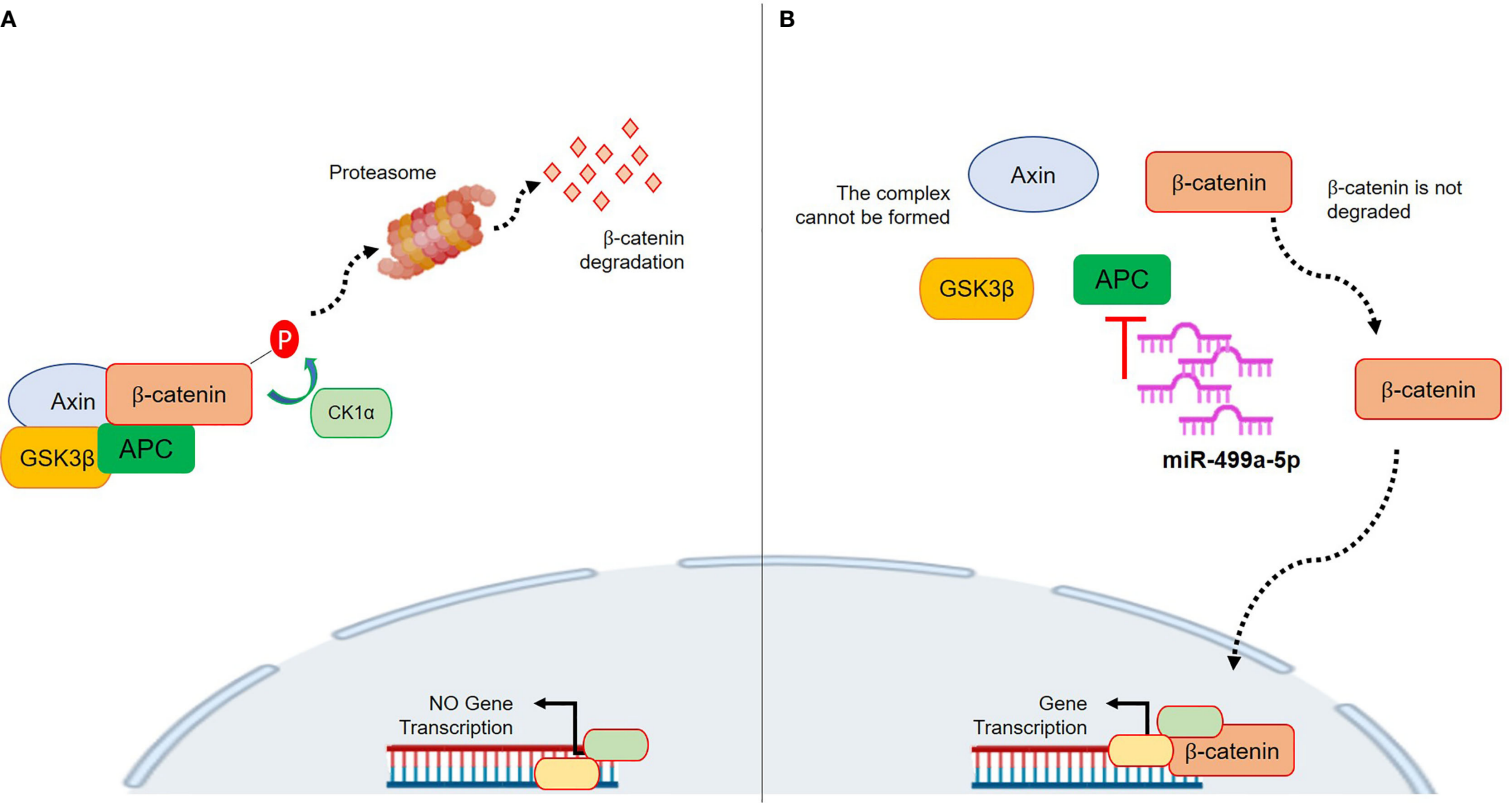
Proposed mechanism of action of miR-499a-5p within the Wnt/β-catenin pathway. **(A)** The complex formed by AXIN, CK1α, GSK3β, and APC phosphorylates β-catenin, which subsequently undergoes the ubiquitin-proteasomal degradation. This prevents its nuclear translocation and pro-tumor gene transcription. **(B)** miR-499a-5p targets APC. High expression of miR-499a-5p promotes APC inhibition, thus preventing the AXIN/CK1α/GSK3β/APC complex formation. Without complex, β-catenin is not degraded, and it can translocate in the nucleus where it promotes gene transcription.

MiR-499a-3p and 499a-5p belong to the miR-499 family, together with miR-499b. Human miR-499 is located in intron 19 of the *MYH7B* gene and it is composed of two genes, miR-499a and miR-499b. Those are located in sense (miR-499a) and anti-sense (miR-499b) DNA chains of the same region and transcribed in antiparallel directions (28). Data about miR-499b are missing, whereas several reports on miR-499a dysregulation have recently been published. Interestingly, a previous work by Zhang et al. showed a link between miR-499a and the Wnt/β-catenin signaling pathway. In particular, the authors demonstrated that overexpression of miR-499a activated β-catenin in cardiac differentiation. With regard to cancer, a few recent studies have tried to clarify the roles of miR-499a. However, it is not surprising that the results were contradictory among the analyzed tumor types because, as well established, the precise function of a single miRNA is strongly dependent on the specific biological context. Li et al. demonstrated that overexpressed miR-499 inhibited non-small cell lung cancer growth by suppressing cell proliferation and promoting apoptosis (29). On the contrary, Liu et al. showed that in colorectal cancer (CRC), miR-499 acts as an onco-mir by targeting two tumor suppressor genes, FOXO4 and PDCD4. Thus, overexpression of miR-499 is associated with advanced CRC stage and with cellular invasion and tumor metastasis in *in vitro* CRC models. In our results, a lower expression of the miRNA was associated with better survival in NSMP patients. Even if a clear mechanism has not been investigated, an interesting explanation could be related to the APC gene. Physiologically, APC acts as a scaffold in the β-catenin destruction complex, so it tightly regulates the Wnt pathway activity (30). APC is a target of miR-499a-5p based on TargetScan prediction, and its upregulation could promote inhibition of APC which, in turn, is less efficient in regulating the β-catenin level (31). Consequently, β-catenin can translocate in the nucleus and activate the Wnt pathway which fosters carcinogenesis. Thus, the higher miR-499a-5p expression could raise the APC inhibition which, consecutively, will be less effective in regulating the cytoplasmatic β-catenin destruction. **Figure 6** shows the proposed mechanism. The CTNNB1 mutations by themselves are classically associated with nuclear translocation and activation of Wnt/β-catenin signaling (15). Interestingly, these mutations are usually found in heterozygosity (17, 32, 33) and, thus, it is reasonable to suppose that miR-499a-5p could play a role in CTNNB1 wild-type cells. Basically, higher miR-499a-5p expression represents an advantage for the tumor cells which may activate transcription of pro-tumorigenic genes besides normal CTNNB1. This could explain why a lower expression of miR-499a-5p resulted to be associated with a better outcome in NSMP patients; however, functional studies are needed in order to test this hypothesis. Our work has several strengths; first, the sample size of NSMP and MMRd patients is numerically important considering the incidence of this tumor. Second, the data were corroborated in the TCGA cohort, an independent and well-described group of EC patients. In the TCGA data, the Illumina platform was used to assess the miRNA expression, whereas we used the RT-PCR-based array and assays; accordingly, two different techniques were exploited to investigate miRNA levels. Third, our cohort was highly homogeneous from a molecular point of view, and this allowed us to limit potential biases; finally, all the patients were homogeneously treated according to ESMO risk classifications. However, there are some limits to consider; among these, we have to include the retrospective design of the study which, however, was essential to creating a model that could be prospectively validated in larger and independent cohorts and possibly on blood samples to assess the validity of the miRNA as a liquid prognostic biomarker. We did not analyze the two miRNAs in the other TCGA groups (i.e., POLE and p53abn groups) because we focused our attention on the NSMP and MMRd ECs in which TCGA had the smallest impact as regards the definition of therapy. Indeed, the classic pathological parameters with all their limitations, defined by ESGO/ESTRO/ESP guidelines, continue to be the gold standard for the therapy selection. Unfortunately, we could not perform any functional validations of our model due to the lack of commercial cell lines with no mutations on p53 or without microsatellite instability.

In conclusion, to the best of our knowledge, we showed, for the first time, that specific miRNAs are associated with the molecular subgroups proposed by the TCGA. miR-499a-5p, in particular, may represent a novel independent prognostic biomarker in NSMP ECs and, if validated in an independent cohort of patients, could improve the current classification and promote a more personalized management of those patients.

## Data Availability Statement

The original contributions presented in the study are included in the article/**Supplementary Material**. Further inquiries can be directed to the corresponding authors.

## Ethics Statement

The studies involving human participants were reviewed and approved by 189/2021/Oss/AOUBo, ClinicalTrials.gov Identifier: NCT04845425. The patients/participants provided their written informed consent to participate in this study.

## Author Contributions

Conceptualization, GR, ADL, and AMP. Methodology, GR, ADL, FG, and DdB. Validation, GR, FG, ADL, and AMP. Formal analysis, GR, CaC, and ADL. Investigation, ADL, DdB, ClC, AMP, and PDI. Resources, AMP and PDI. Data curation, GR, ADL, FG, DdB, AMP, and ClC. Writing—original draft preparation, GR, AMP, ADL, and DdB. Writing—review and editing, GR, CaC, GT, DdB, PDI, AMP, GD, MT, DS, EDC, AGC, and PH. Supervision, GR, AMP, SA, and PDI. All authors contributed to the article and approved the submitted version.

## Funding

The work was supported by Fondazione Cassa di Risparmio in Bologna (Carisbo): project number #19094 to AMP.

## Conflict of Interest

The authors declare that the research was conducted in the absence of any commercial or financial relationships that could be construed as a potential conflict of interest.

## Publisher’s Note

All claims expressed in this article are solely those of the authors and do not necessarily represent those of their affiliated organizations, or those of the publisher, the editors and the reviewers. Any product that may be evaluated in this article, or claim that may be made by its manufacturer, is not guaranteed or endorsed by the publisher.
