# Supplementary material for: Identification of miR-499a-5p as a Potential Novel Biomarker for Risk Stratification in Endometrial Cancer
Source: Front Oncol. 2021 Oct 29;11:757678. doi: 10.3389/fonc.2021.757678 (PMC8597024; doi:10.3389/fonc.2021.757678)
Supplement: Supplementary file 1 [file DataSheet_1.docx]

Supplementary Material

# Supplementary Data

| **Table S1. Characteristics of the study cohort** | | | | |
| --- | --- | --- | --- | --- |
|  | |  | |  |
|  | **Total N=72 (**%) | **NSMP N (%)** | **MMRd N (%)** | **p-value** |
| **Age** | |  |  |  |
| < 50 | 6 (8.3) | 5 (12.2) | 1 (3.2) | 0.22 |
| > 50 | 66 (91.7) | 36 (87.8) | 30 (96.8) |  |
| **BMI** | |  |  |  |
| < 25 | 25 (34.7) | 13 (31.7) | 12 (38.7) | 0.53 |
| > 25 | 47 (65.3) | 28 (68.3) | 19 (61.3) |  |
| **Histotype** | |  |  |  |
| E | 62 (86.1) | 38 (92.7) | 24 (77.4) | 0.14 |
| I-DED | 8 (11.1) | 2 (4.9) | 6 (19.4) |  |
| S | 2 (2.8) | 1 (2.4) | 1 (3.2) |  |
| CS | - | - | - |  |
| CCC | - | - | - |  |
| **ESMO risk** | |  |  |  |
| Low risk | 14 (19.4) | 7 (17.1) | 7 (22.6) |  |
| Intermediate risk | 5 (6.9) | 3 (7.3) | 2 (6.5) |  |
| High-intermediate risk | 31 (43.1) | 22 (53.7) | 9 (29) |  |
| High risk | 22 (30.6) | 9 (22) | 13 (41.9) |  |
| **Grading** | |  |  |  |
| Low | 55 (76.4) | 35 (85.4) | 20 (64.5) | 0.07 |
| High | 17 (23.6) | 6 (14.6) | 11 (35.5) |  |
| **FIGO Stage** | |  |  |  |
| IA | 45 (62.5) | 28 (68.3) | 17 (54.8) | 0.2 |
| IB-II | 13 (18.1) | 8 (19.5) | 5 (16.1) |  |
| III-IV | 14 (19.4) | 5 (12.2) | 9 (29) |  |

**BMI**: Body mass index; **E**: endometrioid; **I-DED**: de-differentiated; **S**: serous; **CS**: carcino-sarcoma; **CCC**: clear cell carcinoma; **MMRd**: MisMatch Repair deficiency; **NSMP**: No specific molecular Profile;

| **Table S2**. List of deregulated miRNAs comparing CTNNB1^mut^ vs CTNNB1^wt^ EC patients in the discovery step | | | | | | |  |
| --- | --- | --- | --- | --- | --- | --- | --- |
| **miRNA** | **P-Value** | **adj.P-Value** | **CTNNB1^mut^ vs CTNNB1^wt^** | | |  |  |
| hsa-miR-499a-5p | 0.000 | 0.0001 | up | | |  |  |
| hsa-miR-499a-3p | 0.000 | 0.0001 | up | | |  |  |
| hsa-miR-516b-5p | 0.001 | 0.073 | up | | |  |  |
| hsa-miR-654-5p | 0.001 | 0.073 | up | | |  |  |
| hsa-miR-325 | 0.002 | 0.090 | down | | |  |  |
| hsa-miR-382-5p | 0.002 | 0.104 | up | | |  |  |
| hsa-miR-187-3p | 0.003 | 0.104 | up | | |  |  |
| hsa-miR-100-5p | 0.003 | 0.104 | up | | |  |  |
| hsa-miR-495-3p | 0.003 | 0.104 | up | | |  |  |
| hsa-miR-455-3p | 0.003 | 0.110 | up | | |  |  |
| hsa-miR-379-5p | 0.004 | 0.117 | up | | |  |  |
| hsa-miR-200a-3p | 0.004 | 0.117 | up | | |  |  |
| hsa-miR-654-3p | 0.007 | 0.160 | up | | |  |  |
| hsa-miR-320a | 0.008 | 0.180 | up | | |  |  |
| hsa-miR-28-3p | 0.009 | 0.180 | up | | |  |  |
| hsa-miR-10a-5p | 0.011 | 0.187 | up | | |  |  |
| hsa-miR-342-5p | 0.011 | 0.187 | up | | |  |  |
| hsa-miR-502-5p | 0.011 | 0.187 | down | | |  |  |
| hsa-miR-31-5p | 0.012 | 0.187 | up | | |  |  |
| hsa-miR-509-3-5p | 0.013 | 0.187 | up | | |  |  |
| hsa-miR-374b-5p | 0.013 | 0.187 | up | | |  |  |
| hsa-miR-369-3p | 0.014 | 0.187 | up | | |  |  |
| hsa-miR-431-5p | 0.015 | 0.201 | up | | |  |  |
| hsa-miR-450a-5p | 0.019 | 0.235 | up | | |  |  |
| hsa-miR-199a-5p | 0.021 | 0.248 | up | | |  |  |
| hsa-miR-485-5p | 0.024 | 0.253 | up | | |  |  |
| hsa-miR-370-3p | 0.026 | 0.253 | up | | |  |  |
| hsa-miR-369-5p | 0.026 | 0.253 | up | | |  |  |
| hsa-miR-487a-3p | 0.026 | 0.253 | up | | |  |  |
| hsa-miR-518e-3p | 0.028 | 0.253 | up | | |  |  |
| hsa-miR-487b-3p | 0.029 | 0.253 | up | | |  |  |
| hsa-miR-193a-5p | 0.030 | 0.253 | up | | |  |  |
| hsa-miR-138-5p | 0.031 | 0.257 | up | | |  |  |
| hsa-miR-486-5p | 0.036 | 0.290 | up | | |  |  |
| hsa-miR-509-5p | 0.041 | 0.296 | up | | |  |  |
| hsa-miR-362-3p | 0.041 | 0.296 | down | | |  |  |
| hsa-miR-597-5p | 0.042 | 0.296 | up | | |  |  |
| hsa-miR-136-5p | 0.043 | 0.296 | up | | |  |  |
| hsa-miR-129-2-3p | 0.045 | 0.306 | up | | |  |  |
|  |  |  |  | | |  |  |
| **Table S3.** Pathway enriched analysis: List of the top 15 pathways predicted by MiRnet tool | | | | | | | |
|  | | | | **P-Value** | **adj.P-Value** | | |
| Gene Expression | | | | 1.18E-08 | 9.10E-07 | | |
| Generic Transcription Pathway | | | | 1.82E-08 | 9.10E-07 | | |
| Signaling by TGF-beta Receptor Complex | | | | 3.45E-07 | 1.15E-05 | | |
| Transcriptional activity of SMAD2/SMAD3:SMAD4 heterotrimer | | | | 1.2E-06 | 0.00003 | | |
| Oncogene Induced Senescence | | | | 2.04E-06 | 4.08E-05 | | |
| Cellular responses to stress | | | | 2.65E-05 | 0.000394 | | |
| SMAD2/SMAD3:SMAD4 heterotrimer regulates transcription | | | | 2.76E-05 | 0.000394 | | |
| PI3K/AKT activation | | | | 3.69E-05 | 0.000461 | | |
| Signaling by VEGF | | | | 4.61E-05 | 0.000512 | | |
| Signaling by Wnt | | | | 7.92E-05 | 0.000792 | | |
| Pre-NOTCH Transcription and Translation | | | | 9.42E-05 | 0.000842 | | |
| Signaling by ERBB4 | | | | 0.000101 | 0.000842 | | |
| G beta:gamma signalling through PI3Kgamma | | | | 0.000118 | 0.000884 | | |
| Constitutive Signaling by AKT1 E17K in Cancer | | | | 0.000172 | 0.000884 | | |
| NGF signalling via TRKA from the plasma membrane | | | | 0.000174 | 0.000884 | | |

## Supplementary Figures


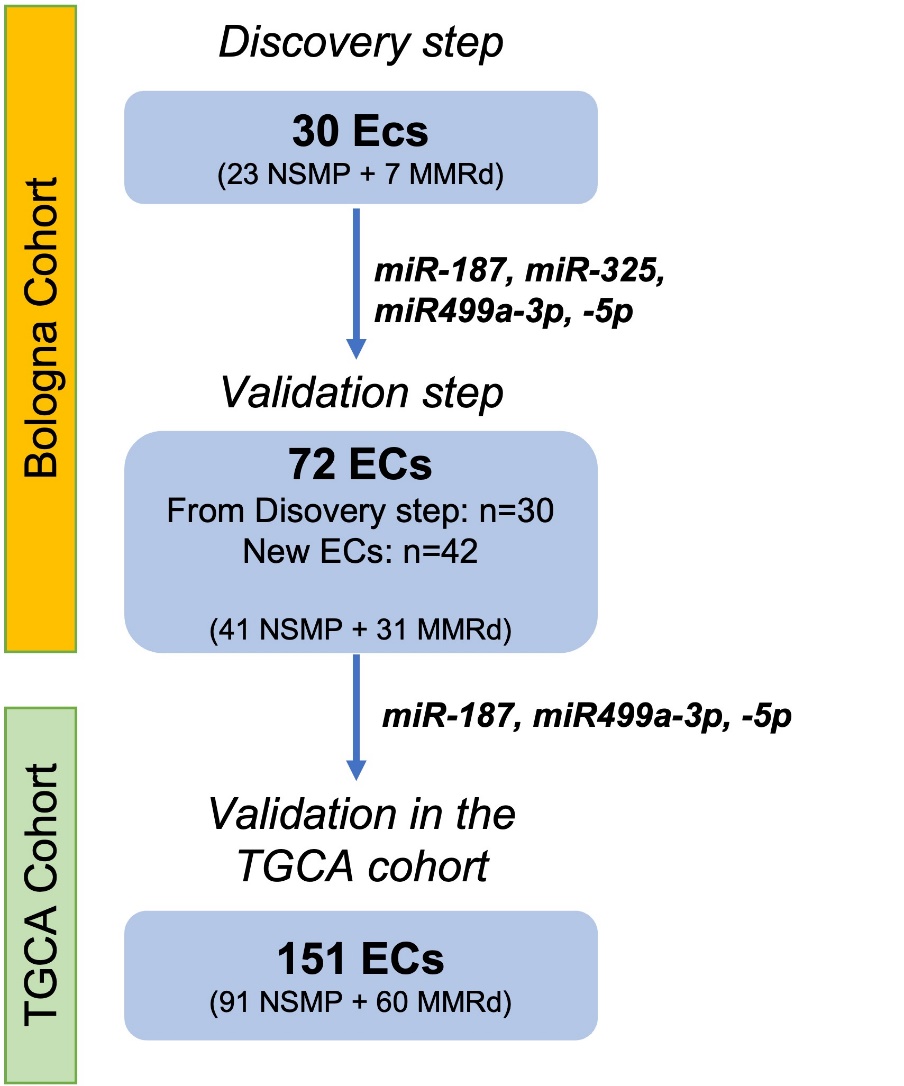


**Supplementary Figure 1**. Workflow of the miRNA analysis


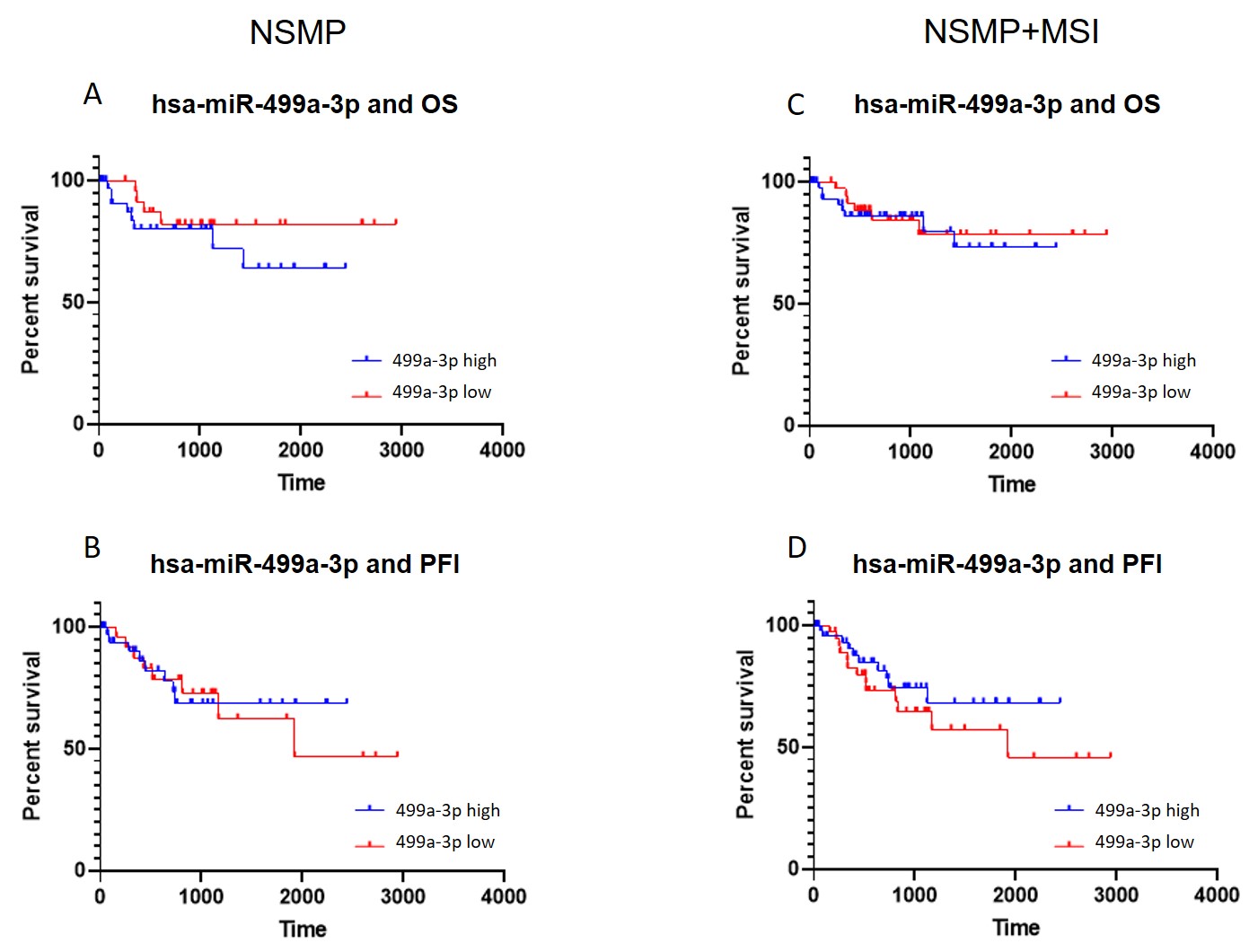


Supplementary Figure 2. Kaplan-Meier estimates of overall survival (panel A and C) and progression free interval (panel B and D) in NSMP alone and NSMP+ MMRd groups, based on miR-499a-3p expression


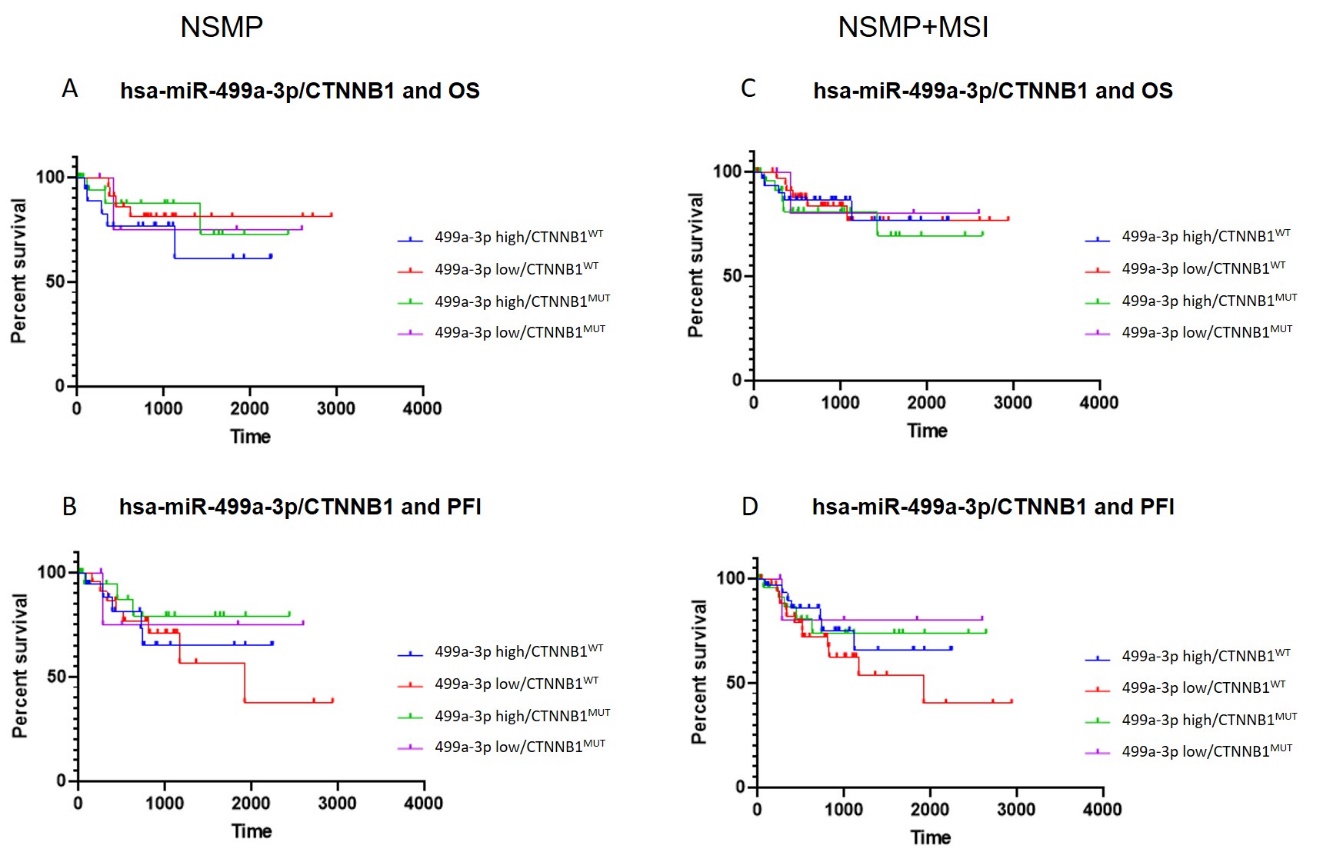


Supplementary Figure 3. Kaplan-Meier estimates of OS (panel A and C) and PFI (panel B and D) in NSMP alone and NSMP+ MMRd groups, based on the combination of miR-499a-3p expression and CTNNB1 status


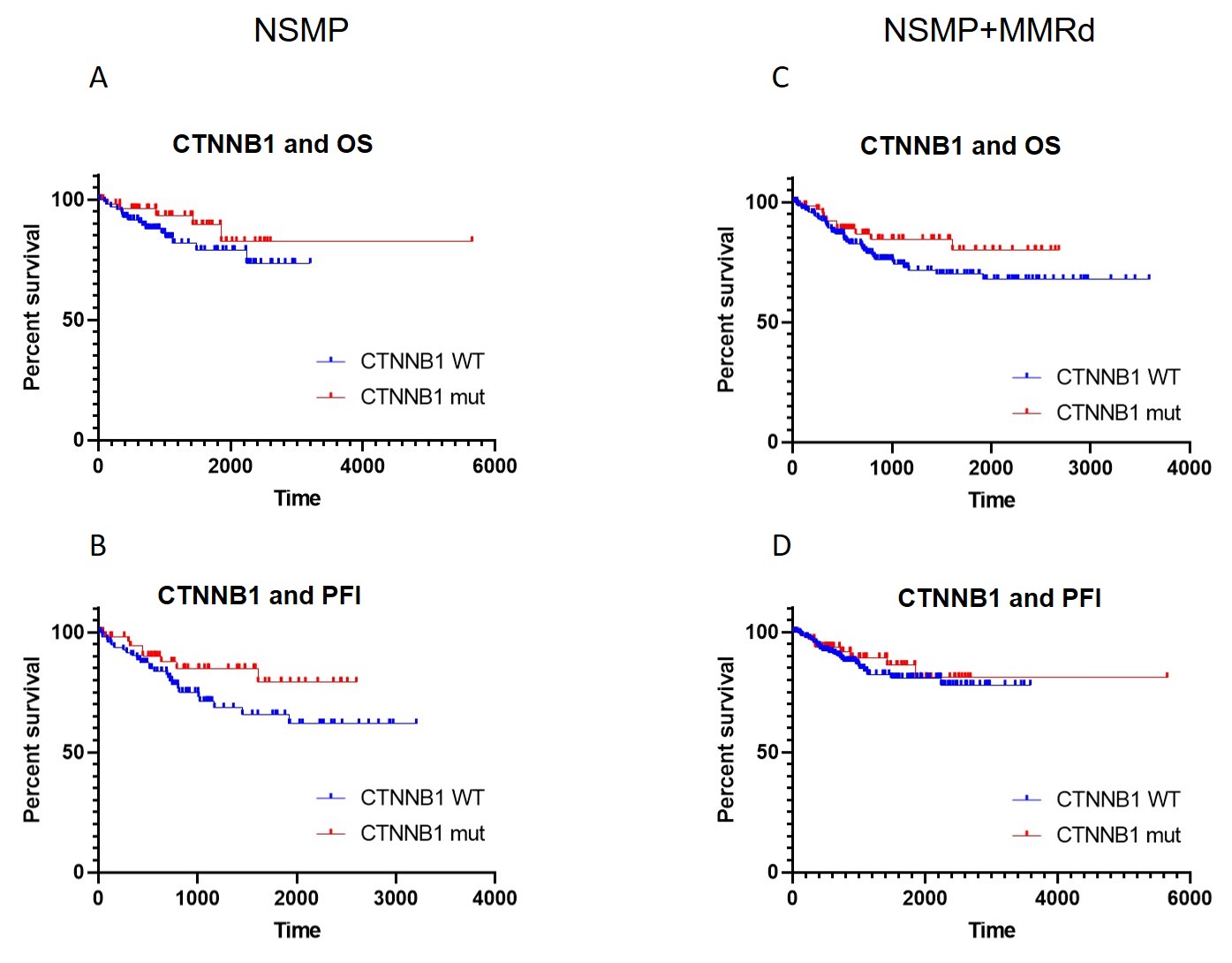


Supplementary Figure 4. Kaplan-Meier estimates of OS (panel A and C) and PFI (panel B and D) in NSMP alone and NSMP+ MMRd groups, based on the CTNNB1 status

**
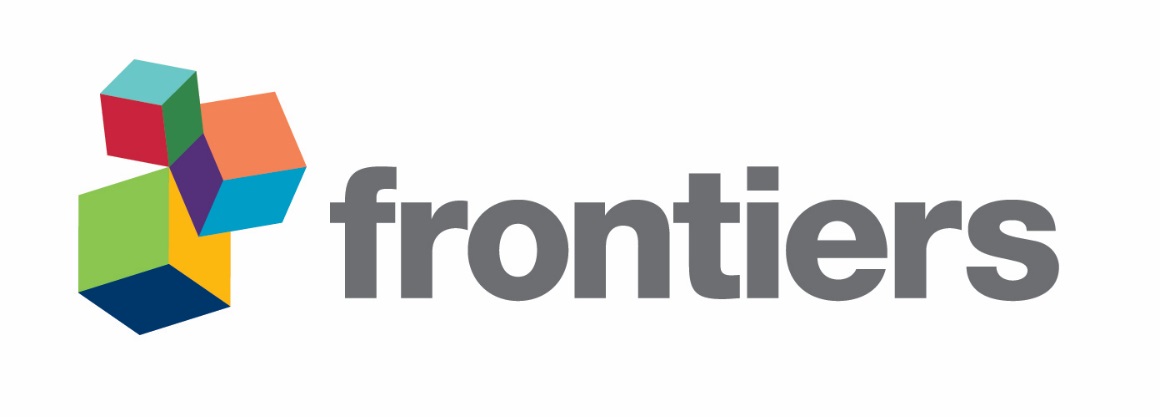
**
